# Synthesis and Fluorescence Mechanism of Nitrogen-Doped Carbon Dots Utilizing Biopolymer and Urea

**DOI:** 10.3390/molecules30092068

**Published:** 2025-05-07

**Authors:** Hikaru Yorozuya, Noor E Ashrafi, Kazuya Sato, Ahatashamul Islam, Rikuto Fukae, Yusuke Tagashira, Toshifumi Iimori

**Affiliations:** Department of Sciences and Informatics, Muroran Institute of Technology, 27-1 Mizumoto-cho, Muroran 050-8585, Japan

**Keywords:** carbon dots, starch, renewable resource

## Abstract

Fluorescent carbon dots are nontoxic nanoparticles composed of carbon, exhibiting advantageous properties for applications in bioimaging and functional materials. We present a methodology for synthesizing fluorescent nitrogen-doped carbon dots (N-CDs) using starch, a biopolymer, and urea as the sources of nitrogen, via the microwave-assisted hydrothermal method. Furthermore, the dependence of the fluorescence spectra and fluorescence quantum yield of N-CDs on the initial concentration of urea in the reactant solution was examined, thereby providing a comprehensive understanding of the influence of nitrogen doping on the CDs. The fluorescence of N-CDs was tunable by varying the excitation wavelength. Stronger fluorescence intensity was observed for a moist phosphate salt/N-CD composite, in contrast to the weaker fluorescence exhibited by a dried one. Fluorescence lifetime measurements revealed that the change in fluorescence intensity can be attributed to the suppression of the non-radiative deactivation process. This observation highlights the critical importance of the interaction between water molecules and surface functional groups in controlling the photophysics of the excited state of N-CDs.

## 1. Introduction

Carbon dots (CDs) are fluorescent nanoparticles that are primarily composed of sp^2^ hybridized carbon and typically exhibit a diameter of less than 10 nm [1]. In the current era of advanced science and technology, CDs have gained traction in diverse fields such as drug delivery and carriers, biological applications, sensing, lighting, catalysis, energy conversion and storage, synthesis, photovoltaics, and capacitors [2,3,4,5,6]. CDs have garnered significant attention from researchers due to their exceptional fluorescence characteristics, excellent biocompatibility, eco-friendly nature, tolerance to photobleaching and photodegradation, ease of bioconjugation, low toxicity [7], and simple synthesis techniques [8] utilizing a broad range of precursors [9]. The physical chemistry of charge transfer in CDs was studied by Guldi et al. [8]. Diverse fluorescence wavelengths are observed for CDs synthesized from similar initial precursors when different synthesis methods are employed [10]. In most cases, the emission wavelength depends on the excitation wavelength; however, fully surface-passivated CDs exhibit excitation-independent emission characteristics [11,12].

Multicolor photoluminescence, an excitation wavelength-dependent emission, and a large Stokes shift are typically observed as the fluorescence characteristics of CDs [13]. In contrast to classical fluorophores, the excitation wavelength dependence of the emission peaks has been widely observed [14,15,16]. Mukherjee et al. prepared N-functionalized carbon nanodots derived from citric acid and urea via the microwave-assisted hydrothermal method [17]. They demonstrated that, in addition to solvent properties such as dipole moments, the photophysical characteristics of the system were controlled by hydrogen bonding. Furthermore, extrinsic parameters such as solvents and dopants significantly influence the emission properties of CDs [15,16]. Specifically, the dipole moment of the polar solvents wherein the carbon nanodots are dispersed plays a crucial role in shifting the fluorescent peak [17].

To enhance the photoluminescence properties of CDs and extend their applicability in biosensing and bioimaging, heteroatom doping is particularly beneficial [18]. Recent studies have demonstrated that heteroatom doping significantly enhances the quantum yield of CDs [19,20]. Nitrogen-doped CDs (N-CDs) have garnered interest due to their promising performance in bioimaging and catalysis applications [13,18,21]. Li et al. demonstrated that N-CDs exhibit tunable fluorescence wavelengths in response to variations in N content [22]. Furthermore, recent research by Dsouza et al. highlighted the significance of surface states in N-CDs, indicating that emissions predominantly originate from surface structures rather than from recombination within the core [23]. Therefore, the surface functional groups play a crucial role in determining the luminescent features of N-CDs.

In this study, N-CDs were synthesized from starch, a biopolymer, and urea via the microwave (MW)-assisted hydrothermal method. The MW-assisted synthesis of CDs has favorable characteristics including a facile synthesis and low energy consumption. In a previous study, CDs were synthesized from starch via MW-assisted hydrothermal synthesis utilizing an aqueous phosphoric acid solution [24]. This prior work involved a detailed investigation into the dependence of the fluorescence properties of CDs to obtain a comprehensive understanding of their formation mechanism. Here, the dependence of fluorescence properties on the quantity of urea was investigated, providing an understanding of the impact of nitrogen doping on the fluorescence characteristics of CDs, which remains poorly understood. Furthermore, the influence of water on the fluorescence properties of N-CDs remains an open question. In this study, the fluorescence enhancement of N-CDs upon interaction with water was demonstrated. This study demonstrates that N-CDs are potential candidates for fluorescence bioimaging, offering safer and cost-effective alternatives to toxic and expensive quantum dots.

## 2. Results and Discussion

### 2.1. Fluorescence in Aqueous Phosphoric Acid Solution

**Figure 1:**
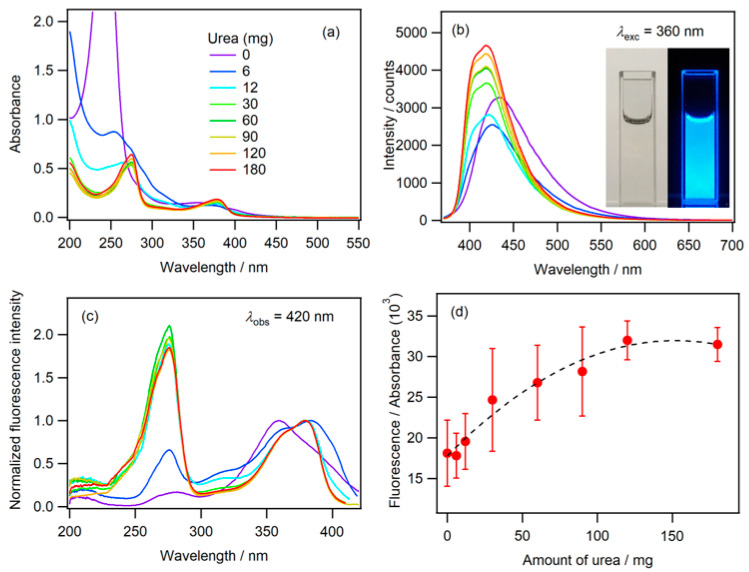
(**a**–**c**) Absorption, fluorescence, and fluorescence excitation spectra of the N-CDs synthesized using varying amounts of urea in phosphoric acid aqueous solution. The color code indicating the amount of urea is common to (**a**–**c**). (**d**) The fluorescence intensity divided by the absorbance at the excitation wavelength versus the amount of urea. The value on the vertical axis represents a relative measure of the fluorescence QY. (Inset) Images of the N-CDs in solution under (left) ambient light, and (right) excitation at 365 nm. λ_exc_ and λ_obs_ represent the excitation and observed wavelengths, respectively.

The absorption spectra of N-CDs prepared using varying amounts of urea are presented in Figure 1a. The N-CDs prepared using x mg of urea are denoted as N-CDs(Ux) hereafter. The synthesis procedure of N-CDs is described in Section 3. Briefly, the N-CDs were obtained from the MW-assisted hydrothermal reaction of starch and urea in an aqueous phosphoric acid solution. As the N-CDs were synthesized using this reaction scheme, they were obtained in the dispersed state in an aqueous phosphoric acid solution. The spectra in Figure 1 were measured using N-CDs dispersed in an aqueous phosphoric acid solution in their as-prepared state. A sharp absorption band with a maximum at 244 nm was observed for CDs(U0). A redshift in the absorption maximum was observed as the amount of urea increased from 0 to 30 mg. N-CDs prepared with more than 30 mg of urea exhibited an absorption maximum at 275 nm and an absorption band in the range of 320–450 nm. The absorption spectrum of CDs(U0) exhibited an absorption tail at wavelengths longer than 400 nm. The absorption band of N-CDs prepared using the reactant solution containing urea exhibited a narrower bandwidth than that of CDs(U0). Two absorption bands with maxima at 275 and 379 nm were observed for N-CDs(U120) and N-CDs(U180). These absorption bands were attributed to the π–π* transition of the sp^2^-conjugated system, the transition of π electrons in surface states, and the transition from the non-bonding orbital on heteroatoms or functional groups such as C=O to the π* orbital of the carbon double bonds [25,26,27].

Blue fluorescence was observed with photoexcitation of the N-CDs (Figure 1). The fluorescence and fluorescence excitation spectra are shown in Figure 1b,c, respectively. The maximum fluorescence of the CDs(U0) was observed at 435 nm. For the N-CDs prepared using urea, the fluorescence maximum was blue shifted to 419 nm, and the width of the fluorescence spectrum narrowed as the amount of urea increased. The fluorescence excitation spectra of the N-CDs prepared using more than 6 mg of urea exhibited maxima at 275 and 379 nm, which were consistent with the absorption spectrum.

The surface states originating from functional groups containing oxygen, which may be derived from starch, acid, and solvent, have been proposed to affect the fluorescence characteristics of CDs [28]. The nitrogen doping of CDs can lead to the generation of defect sites in the sp^2^-conjugated system of carbon atoms within the graphite structure, in addition to introducing surface states originating from functional groups containing nitrogen [27]. The fluorescence intensity divided by the absorbance as a function of urea concentration is depicted in Figure 1d. The fluorescence intensity is proportional to the product of the fluorescence quantum yield (QY) and the absorbance of the sample [29]. Therefore, the relative magnitude of the fluorescence QY for N-CDs synthesized using varying urea concentrations can be evaluated from this figure. It is reasonable to assume that changes in the structure, such as the degree of N-doping of the CDs and the density of the nitrogen-containing functional groups on the surface of the CDs, occur as the urea concentration increases. The results presented in Figure 1d indicate that the fluorescence QY increased as the degree of N-doping to the carbon framework and the density of the surface functional groups incorporating nitrogen increased.

**Figure 2:**
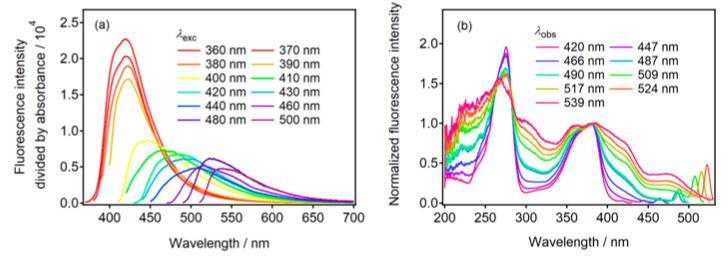
(**a**) Fluorescence spectra of N-CDs(U90) observed at different excitation wavelengths (λ_exc_). The fluorescence intensity was divided by the absorbance at λ_exc_. (**b**) Fluorescence excitation spectra at various observed wavelengths. The fluorescence intensity in (**b**) was normalized at approximately 380 nm.

The dependence of fluorescence on the excitation wavelength is illustrated in Figure 2a. The fluorescence maximum shifted to longer wavelengths as the excitation wavelength increased. The fluorescence maximum was observed at 538 nm with an excitation at 500 nm. This result demonstrates that N-CDs exhibit multicolor fluorescence properties, and that the fluorescence color can be tuned by selecting the wavelength of the excitation light. Fluorescence excitation spectra were measured to gain insights into the origin of the multicolor fluorescence (Figure 2b). The narrow bands that were observed in the range of 450 to 530 nm with λ_obs_ > 466 nm were attributed to the Raman scattering of the aqueous phosphoric acid solution. When fluorescence was monitored at 420 nm, two excitation bands were observed at 275 and 379 nm. The widths of the excitation bands broadened as the observed wavelength increased. The fluorescence excitation spectrum, obtained by observing fluorescence longer than 509 nm, indicated an additional absorption band at approximately 475 nm. N-CDs exhibiting broad absorption bands emitted fluorescence at long wavelengths. These changes in the excitation band characteristics indicate that the sample contained N-CDs with varying compositions. This difference in composition was attributed to the different degrees of doping by heteroatoms in the sp^2^-conjugated system of the graphite structure. Additionally, the heterogeneous distribution of the functional groups on the surface of the N-CDs may contribute to this variability. The structural diversity within N-CDs results in differences in their fluorescence properties.

### 2.2. Fluorescence of Phosphate Salt Containing N-CDs

The optimal pH range for aqueous solutions dispersing fluorescent N-CDs for potential applications in biological imaging lies between pH = 6 and 8 [30,31,32]. In this study, N-CDs dispersed in an aqueous phosphoric acid solution were obtained via MW-assisted synthesis. We hypothesized that phosphate salts synthesized from an aqueous phosphoric acid solution dispersing N-CDs could provide water-soluble N-CDs suitable for biological imaging applications.

**Figure 3:**
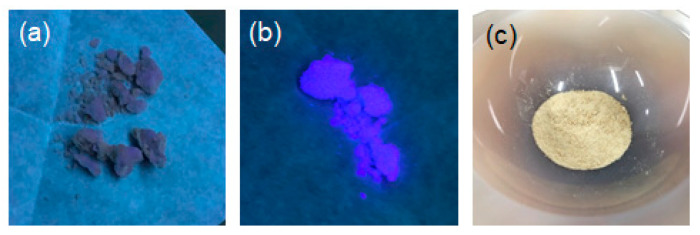
Images of (**a**) the dried and (**b**) moist phosphate salt/N-CDs(U120) composites observed when excited at 365 nm. The powder of the composite was placed on weighing paper. (**c**) Image of the composite placed on an agate mortar after grinding, observed under ambient light.

**Figure 4:**
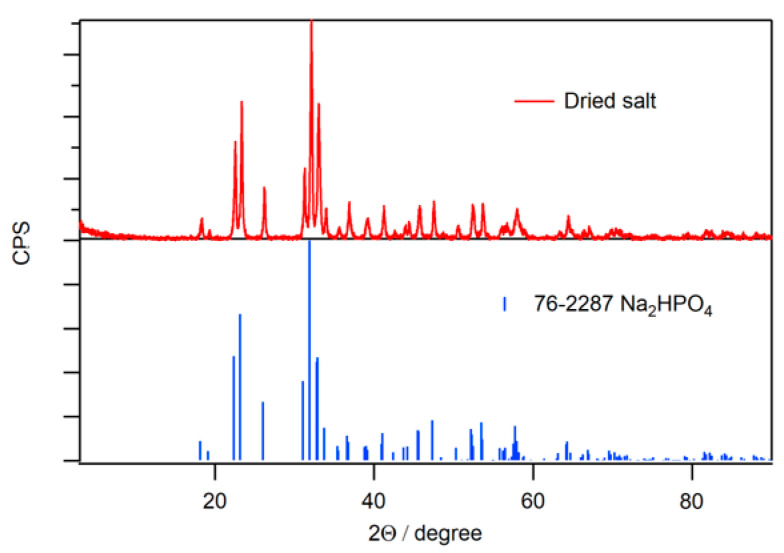
(**Top**) XRD pattern of the dried phosphate salt/N-CD composite. (**Bottom**) Reference pattern of Na_2_HPO_4_ (PDF 76-2287).

A sediment of the solid phosphate salt/N-CD composite was formed following the addition of an aqueous NaOH solution to an aqueous phosphoric acid solution containing the N-CDs. The phosphate salt was collected via filtration and subsequently dried in vacuum for 24 h. After grinding the dried phosphate salt in an agate mortar, a brownish powder was obtained (Figure 3). This powder was analyzed via powder X-ray diffraction (XRD) (Figure 4) and identified as Na_2_HPO_4_.

**Figure 5:**
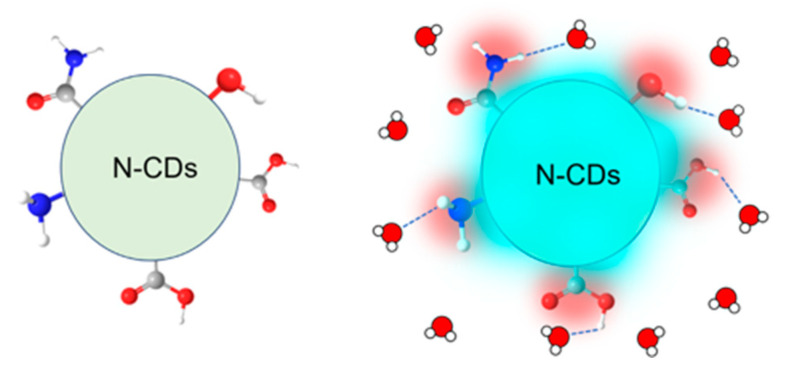
(**Left**) N-CDs in a dried condition showing a lower fluorescence intensity. (**Right**) Fluorescence enhancement of N-CDs upon binding water molecules to the functional groups that exist on the surface of N-CDs. Water molecules are represented by one red and two white balls.

The fluorescence intensity increased as the moisture content in the powder of the phosphate salt/N-CD composite increased. The moist composite exhibited blue fluorescence with the photoexcitation at 365 nm (Figure 3). In contrast, the dried salt exhibited very weak fluorescence. This switching of fluorescence properties occurred as the moisture content of the composite varied, indicating that the surface state of the N-CDs and the presence of water are critical factors in N-CD fluorescence (Figure 5).

**Figure 6:**
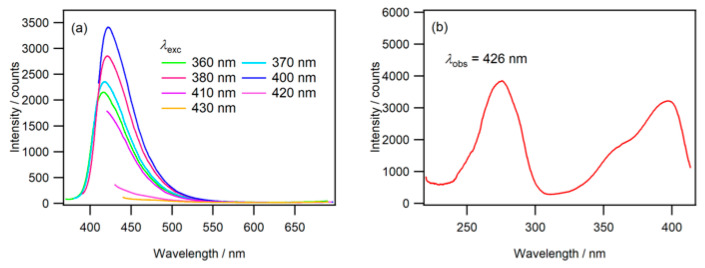
(**a**) Fluorescence spectra of the moist phosphate salt/N-CDs(U120) composite. (**b**) Fluorescence excitation spectrum observed at wavelength of 426 nm.

The fluorescence spectra of the moist phosphate salt/N-CDs(U120) composite are presented in Figure 6a. The fluorescence maximum was observed at 422 nm when excited at 400 nm, which closely aligned with that observed for N-CDs(U120) in an aqueous phosphoric acid solution. Additionally, the fluorescence excitation spectrum of the moist salt (Figure 6b) retained the characteristics observed for N-CDs(U120) in an aqueous phosphoric acid solution.

**Figure 7:**
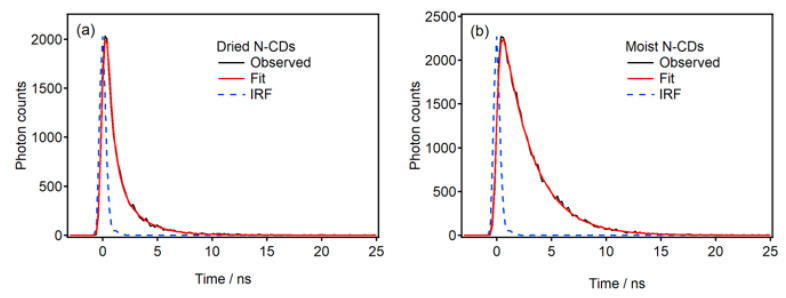
Fluorescence decay curves (black) and the results of the fitting (red) using the instrument response function (IRF) (blue) for (**a**) the dried and (**b**) moist phosphate salt/N-CD composites. The excitation and observed wavelengths were 405 and 422 nm, respectively.

The insight into the mechanism of the fluorescence in the N-CDs was studied using the fluorescence lifetime measurements. The fluorescence decay curves of the dried and moist composites are shown in Figure 7. A multi-exponential decay function was used in the nonlinear least-squares fitting of the decay curves:

(1) $$I\left(t\right)=\sum_{i=1}^{2}A_{i}\exp\left(-\frac{t}{\tau_{i}}\right),\;$$

where *I*(*t*) is the fluorescence intensity as a function of time *t*, *A_i_* is a pre-exponential factor, and *τ_i_* is the lifetime of the *i*-th component. The result of the fitting is summarized in Table 1. The fluorescence decay curve for the moist composite was fitted using a single-component exponential function. The fluorescence QY is given by the ratio of $k_{R}/(k_{R}+k_{NR})$, where $k_{R}$ and $k_{NR}$ are the radiative and non-radiative rate constants of the excited state, respectively. The fluorescence lifetime *τ* is given by ${\tau =(k_{R}+k_{NR})}^{-1}$. The longer fluorescence lifetime of the moist composite than that of the dried one demonstrates that the non-radiative rate constant $k_{NR}$, which corresponds to the deactivation rate in the excited state of N-CDs, is reduced by the binding of water molecules to the surface of N-CDs. The increase in the fluorescence QY and fluorescence intensity for the moist composite (Figure 3) can be reasonably explained by the decrease in the $k_{NR}$.

### 2.3. Fluorescence of N-CDs in Neutral Aqueous Solution

**Figure 8:**
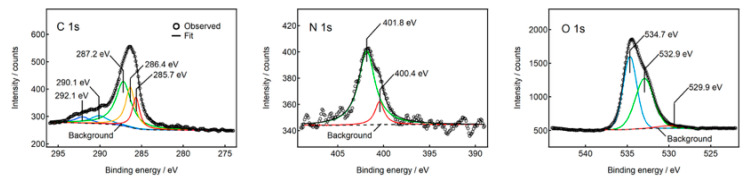
XPS spectra of carbon, nitrogen, and oxygen atoms of N-CDs(U120).

The N-CDs were isolated from the phosphate salt by dissolving the Na_2_HPO_4_/N-CD composite in distilled water, followed by desalting using dialysis. A brownish sediment was formed after 78 h of dialysis. X-ray photoelectron spectroscopy (XPS) was employed to confirm the nitrogen doping to the CDs. The desalted and isolated N-CDs(U120) were used as the sample. The XPS spectra of the carbon, nitrogen, and oxygen 1s peaks are shown in Figure 8. The nitrogen 1s peak was clearly observed, confirming that nitrogen was covalently conjugated to the CD framework. The relative atomic fraction of carbon and nitrogen evaluated from the peak intensity was 89 and 11%, respectively.

**Figure 9:**
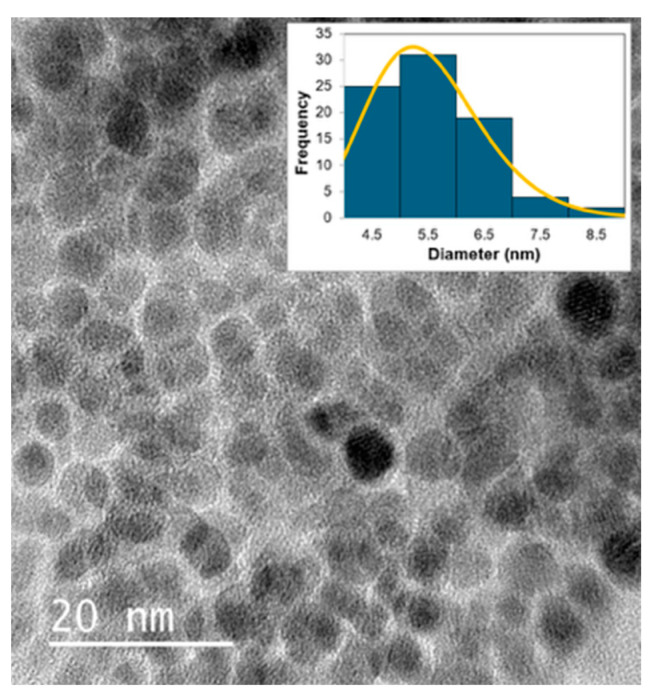
TEM image of the N-CDs. The inset shows the histogram (blue bars) of the distribution of the diameter of N-CDs, and the fitting result (orange) of the distribution obtained by assuming the log-normal distribution function of the diameter.

The morphology of the isolated N-CDs was characterized using transmission electron microscopy (TEM) measurements. A drop of the N-CD dispersion in water, which was subjected to ultrasonic treatment, was employed to prepare the specimen for the TEM measurements. The TEM image of the N-CDs is shown in Figure 9. The histogram of the particle diameter is shown in the inset of Figure 9. The distribution of the diameters of nanoparticles is known to follow the log-normal distribution function [33]. The result of the fitting obtained by assuming the log normal distribution function of the diameters is also shown in Figure 9. The average particle diameter was obtained as 5.5 nm from the fitting parameters in the log-normal distribution function. The d-spacing of the nanoparticles was approximately 0.215 nm, which is consistent with the d-spacing observed in graphite. This result validated that the nanoparticles observed were CDs.

**Figure 10:**
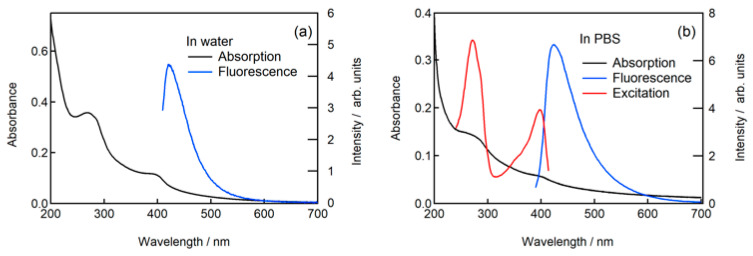
(**a**) Absorption and fluorescence spectra of desalted and isolated N-CDs dispersed in water. The fluorescence was observed with the excitation at 400 nm. (**b**) Absorption, fluorescence, and fluorescence excitation spectra of N-CDs in PBS solution. The fluorescence and fluorescence excitation spectra were observed with the excitation at 380 and 424 nm, respectively.

An aqueous dispersion of N-CDs(U120) was obtained by subjecting the mixture of the desalted N-CDs and distilled water to ultrasonic treatment for 25 min. The absorption and fluorescence spectra of the N-CDs are shown in Figure 10a. The fluorescence QY was obtained as 7.60% with the excitation at 390 nm. The absorption band maxima were observed at 277 and 400 nm. The fluorescence peak was observed at 424 nm when excited at 400 nm.

### 2.4. Fluorescence of N-CDs in Phosphate Buffered Saline

The desalted and isolated N-CDs(U120) were dispersed in phosphate buffered saline (PBS) via ultrasonic treatment for 10 min. The absorption bands with the maxima at 262 and 403 nm were observed in the absorption spectrum (Figure 10b). The fluorescence spectrum had a fluorescence peak at 424 nm with excitation at 380 nm (Figure 10b), which closely aligned with the fluorescence spectrum of N-CDs(U120) in an aqueous phosphoric acid solution (Figure 1). Furthermore, the fluorescence excitation spectrum in PBS also exhibited features analogous to those of N-CDs(U120) in an aqueous phosphoric acid solution (Figure 1), as illustrated in Figure 10b. These results indicate that the optical properties of N-CDs(U120) remain unchanged even after dissolution in PBS, indicating their potential suitability for diverse biomedical and bioanalytical applications.

### 2.5. Formation Mechanism of N-CDs

Starch, utilized as the carbon source for CD synthesis in the present study, is a polysaccharide made up of repeating glucose units. Numerous investigations have explored the use of starch as a precursor for the fabrication of carbon dots [24,34,35,36,37,38,39,40,41,42]. Despite extensive research on the synthesis of CDs from carbohydrates, the precise synthetic pathway remains elusive due to the presence of numerous intermediates, many of which are challenging to identify in practice [24,43]. One proposed mechanism suggests that the formation of CDs initiates with the hydrolysis of starch in an aqueous phosphoric acid solution [24]. Phosphoric acid may function as an agent of the reaction of polysaccharide [43]. Starch is converted to glucose and other saccharides by hydrolysis in the presence of H^+^ under hydrothermal condition [44]. Glucose and other saccharides undergo dehydration-induced degradation, leading to the formation of furfural intermediates [45]. These intermediates may subsequently polymerize into aromatic structures, which eventually condense into a carbonaceous material [43,45]. The dehydration of these chemical species has also been proposed as a pathway to the formation of polymers containing aromatic rings and condensed carbonaceous materials [46,47,48]. The carbon-containing molecules formed through these processes ultimately become CDs. Reactive chemical species containing nitrogen may be formed when urea is reacted at high temperature in the presence of phosphoric acid. Although the formation mechanism of N-CDs is not fully understood yet [49,50], the nitrogen atoms derived from urea can be incorporated into the sp^2^ hybrid orbital of the carbon framework and can also contribute to the formation of the amine functional groups.

## 3. Materials and Methods

### 3.1. Materials

Commercially available starch derived from potatoes harvested in Hokkaido, Japan (Kawamitsu Bussan Co., Ltd., Tokyo, Japan) was utilized. Urea (Wako Pure Chemicals, Osaka, Japan), phosphoric acid (85%, Junsei Chemical, Tokyo, Japan), and NaOH (Junsei Chemical) were utilized as received, without further purification.

### 3.2. Synthesis Procedure

Starch was mixed with 26 mL of distilled water in a glass flask and stirred for 15 min by employing a magnetic stirring bar. A specific amount of urea and 4 mL of phosphoric acid were subsequently added to the aqueous starch solution. The solution was stirred and heated in a microwave oven (Panasonic, Japan, 250 W) for 12 min. During heating, the mouth of the flask was sealed with a perforated Kapton^®^ film. The solution was cooled to room temperature, followed by the addition of distilled water (20 mL) and subsequent stirring. The resulting solution was filtered sequentially employing filter paper (Advantec, Japan), followed by polytetrafluoroethylene membrane filters with pore sizes of 1.0 μm (Merck, Germany) and 0.2 μm (Advantec). The phosphate salt was precipitated by neutralizing an aqueous phosphoric acid solution containing CDs with 5 M aqueous NaOH. The aqueous solution of the phosphate salt was dialyzed for 78 h employing a Pur-A-Lyzer^TM^ dialysis tube with a molecular wight cutoff of 1 kDa (Merck, Germany). The yield of the dialyzed N-CDs was 7% by weight.

### 3.3. Characterization Methods

The absorption spectra were recorded with a spectrophotometer (Hitachi, Tokyo, Japan, U4100). The fluorescence spectra were measured with a spectrofluorometer (Hitachi, F4500). The solution was placed in a quartz cell with an optical path length of 1 cm for both the absorption and fluorescence measurements. TEM (JEOL, Tokyo, Japan, JEM-2100F), equipped with a field emission gun at an acceleration voltage of 200 kV, was employed to observe the N-CD morphology. The TEM specimen was prepared by placing a drop of a solution containing N-CDs onto a Cu grid with a carbon support film. XRD was performed by employing an X-ray diffractometer (Rigaku, Tokyo, Japan, MultiFlex-120NP) utilizing a Cu Kα X-ray source (1.54058 Å) operated at 40 kV and 20 mA. The fluorescence QY was measured relative to a fluorescence standard, unless otherwise noted [29]. Quinine sulfate dissolved in 0.1 mol/L aqueous H_2_SO_4_ served as the standard solution. The excitation wavelength of both the standard solution and N-CDs dispersed in an aqueous solution of phosphoric acid was 350 nm. The absorbance of the standard solution at 350 nm was adjusted to 0.05. The fluorescence QY was calculated using a QY value of 0.577 for the standard solution [29]. The refractive indices of aqueous solutions of H_2_SO_4_ and phosphoric acid were assumed to be identical to those of water. An X-ray photoelectron spectrometer (JEOL, JPS-9010MX), equipped with a Mg Kα radiation source operated at 10 kV and 10 mA, was used to record the XPS spectra of the N-CDs. The satellite peaks were subtracted from the observed spectra. The fluorescence decay curves were measured using a time-correlated single photon counting technique (Hamamatsu Photonics, Hamamatsu, Japan, Quantaurus Tau).

## 4. Conclusions

N-CDs dispersed in aqueous phosphoric acid solution were synthesized using the MW-assisted hydrothermal method, wherein starch (a biopolymer) and urea were employed as the carbon and nitrogen source, respectively. A detailed investigation of the optical properties of the synthesized N-CDs was conducted by varying the urea concentration. The CDs that were synthesized without urea had an absorption band at 244 nm. The absorption spectra of N-CDs synthesized using urea exhibited a red shift in the absorption band, and two absorption bands with maxima at 275 and 379 nm were observed for N-CDs(U120). In contrast, the fluorescence maximum at 430 nm underwent a blue shift, in addition to a narrower fluorescence band, with the increase in urea quantity, indicating a significant change in fluorescence characteristics resulting from the increased nitrogen content. The fluorescence QY indicated a direct correlation with both the degree of doping and the density of the surface functional groups. The measured fluorescence QY was 7.6% for N-CDs(U120) in water. A detailed investigation of the dependence of fluorescence characteristics on the excitation wavelength indicated multicolor fluorescence when excited at different wavelengths. The fluorescence intensity of the phosphate salt/N-CD composite markedly increased with the increase in the water content, demonstrating that both water molecules and the surface functional groups of N-CDs have a significant influence on the non-radiative deactivation process in the excited state. The synthesis procedure for N-CDs, along with their diverse properties and the underlying mechanisms for these characteristics, presents potential for various applications.

## Figures and Tables

**Table 1:** Fluorescence lifetime of the dried and moist phosphate salt/N-CD composites. The pre-exponential factors of the biexponential decay function are shown in parentheses. The fluorescence decay curve for the moist composite was fitted using a single-component exponential function. $\bar{\tau }$ is the average fluorescence lifetime.

	$\tau_{1}$	$\tau_{2}$	$\bar{\tau }$
Dried	0.42 ns (0.778)	2.0 ns (0.222)	1.3 ns
Moist	2.7 ns	-	2.7 ns

## Data Availability

The original contributions presented in this study are included in the article material. Further inquiries can be directed to the corresponding author.
